# Isolation and Characterization of Hydrocarbon-Degrading Yeast Strains from Petroleum Contaminated Industrial Wastewater

**DOI:** 10.1155/2015/929424

**Published:** 2015-08-03

**Authors:** Boutheina Gargouri, Najla Mhiri, Fatma Karray, Fathi Aloui, Sami Sayadi

**Affiliations:** Laboratoire des Bioprocédés Environnementaux, Centre de Biotechnologie de Sfax, Université of Sfax, BP 1177, 3018 Sfax, Tunisia

## Abstract

Two yeast strains are enriched and isolated from industrial refinery wastewater. These strains were observed for their ability to utilize several classes of petroleum hydrocarbons substrates, such as* n*-alkanes and aromatic hydrocarbons as a sole carbon source. Phylogenetic analysis based on the D1/D2 variable domain and the ITS-region sequences indicated that strains HC1 and HC4 were members of the genera* Candida* and* Trichosporon*, respectively. The mechanism of hydrocarbon uptaking by yeast,* Candida, *and* Trichosporon* has been studied by means of the kinetic analysis of hydrocarbons-degrading yeasts growth and substrate assimilation. Biodegradation capacity and biomass quantity were daily measured during twelve days by gravimetric analysis and gas chromatography coupled with mass spectrometry techniques. Removal of* n*-alkanes indicated a strong ability of hydrocarbon biodegradation by the isolated yeast strains. These two strains grew on long-chain* n*-alkane, diesel oil, and crude oil but failed to grow on short-chain* n-*alkane and aromatic hydrocarbons. Growth measurement attributes of the isolates, using* n*-hexadecane, diesel oil, and crude oil as substrates, showed that strain HC1 had better degradation for hydrocarbon substrates than strain HC4. In conclusion, these yeast strains can be useful for the bioremediation process and decreasing petroleum pollution in wastewater contaminated with petroleum hydrocarbons.

## 1. Introduction

Soil and water contamination are frequently caused by oil and oil-related compounds. Strategies for controlling environmental contamination by petroleum and its derivatives have been the subject of various studies over the past three decades. When a spillage occurs, the first action is to remove the oily phase by mechanical or physicochemical means through the application of surfactants in order to disperse the oil layer. Although a variety of physicochemical techniques are available for the clean-up of water surface, some interest in the use of microbial biodegradative activity is growing [1]. Biodegradation is an alternative that has been used to eliminate or minimise the effects of pollutants by using microorganisms which have biodegradation potential [2]. In these environments, organic pollutants frequently occur when mixed with other synthetic or natural organic compounds. Microorganisms, potentiality pointed out in the literature as degradation agents of several compounds, indicate that biodegradation is one of the most promising alternative of biological treatments to reduce the environmental impact caused by oil spills. Moreover, it is known that the main microorganisms consuming petroleum hydrocarbons are bacteria, yeasts, and fungi [3].

Therefore, it is necessary to understand how the biodegradation of the polluting compounds is affected by the presence of alternate substrates. It is also necessary to study the microbial degradation of crude oil as an environmentally friendly way of cleaning up oil-polluted areas.

The first step in such studies is to isolate and identify the microorganisms from contaminated soil and water which are capable of crude oil degradation. Among many studies conducted on microbial biodegradation of oil-related contaminants, more than 80% are devoted to bacterial biodegradation. Bacteria are the most studied microorganisms and their participation during hydrocarbon mineralization in water has been studied by many authors [4]. However, not only are bacteria capable of hydrocarbon biodegradation but also yeasts isolated from hydrocarbon contaminated sites display the ability to use oil-related compounds [5, 6]. As a matter of fact, a wide variety of yeasts and filamentous fungi are capable of utilizing hydrocarbons. Yeasts capable of degrading hydrocarbons include genera of* Yarrowia lipolytica*,* Candida tropicalis*,* Candida albicans,* and* Debaryomyces hansenii*. The alkane-utilizing yeast* Y. lipolytica* degrades very efficiently hydrophobic substrates (HS) such as triglycerides (fats, oils), alkanes, and fatty acids. It has been observed that* Y. lipolytica* is the predominant yeast form during alkane degradation and emulsifier production [7].

The study of hydrocarbon biodegradation by native microorganisms is of extreme ecological importance. The actual contribution of yeasts in the biodegradation of hydrocarbons in the environment may be more important than that previously expected, considering the metabolic diversity demonstrated for yeasts [8]. However, bioavailability of hydrophobic organic compounds to microorganisms is generally a limiting step during the biodegradation process [9]. Hydrophobic interactions play an important role in the adherence of microorganisms to a wide variety of surfaces [10]. In particular, the hydrophobic nature of the bacterial surface has been cited as a key factor in cells growth on water-insoluble hydrophobic substrates such as hydrocarbon [11, 12]. To enter cells, HS must interact with the cell surface. Two hypotheses have been formulated to explain this step of poorly water-miscible substrates transport into microorganisms: (i) the compounds can be solubilised (or pseudosolubilised) in the presence of surface-active compounds (surfactant-mediated transport) or (ii) they can adhere directly to the cell wall (direct interfacial transport) [13]. The aforementioned yeast species can produce surfactants during growth on HS. A correlation has been observed between cells and HS adhesion induction and the increase in apolar properties of the cell surface [13]. Aerobic degradation of alkanes can be carried out by two major types of enzymes: the alkane monooxygenase (also known as alkane hydroxylase, or AlkB) and certain cytochrome P450 systems.

In a screening program carried out in our laboratory, we isolated yeast strains from petroleum contaminated wastewater effluents that are able to utilize hydrocarbons. The use of naturally occurring yeasts represents a potentially promising means of destroying polluting chemicals in wastewater treatment systems and soil. Therefore, the objective of the present work was to isolate, identify, and characterize* n*-alkane using yeasts capable of degrading petroleum hydrocarbons in order to employ yeasts strains in the future as a biodegrader of polluted environment. The hydrocarbon degradation activity and cell hydrophobicity were also investigated in relation to* n*-alkane utilization.

## 2. Material and Methods

### 2.1. Isolation and Characterization of Yeast Isolates

The hydrocarbons-degrading microbial consortium was enriched from petrochemical industrial wastewater using a continuously stirred tank-reactor CSTR [14]. Two of the best-growing yeast strains were isolated from the hydrocarbons-degrading microbial consortium. The yeast strains were isolated by using a selective enrichment culture and a single-colony isolation technique. The selected yeasts were identified in the present study by routine morphological, microbiological, and biochemical methods following directions of the latest edition of Bergey's Manual [15].

### 2.2. Culture Conditions

Yeast isolates were cultivated in 250 ml Erlenmeyer flasks containing 50 ml of mineral medium (MM). The pH was measured using the Cyberscan 500 pH meter. The pH was adjusted by using 1 N standard NaOH and 1 N standard HCl (Merck, Germany). The mineral medium used contained per litre 3.4 g K_2_HPO_4_, 4.3 g KH_2_PO_4_, 0.3 g MgCl_2_·2H_2_O, and 1 g (NH_4_)_2_SO_4_, which was supplemented with 1 ml of a trace element solution [16]. The media used for the yeast growth were sterilized by autoclaving at 121°C for 20 minutes. Petroleum compounds and derivatives were added as carbon source to the sterilized MM at the desired concentrations. A liquid culture was started by adding a loop full of cells from a standard agar plate into a 250 ml Erlenmeyer flask containing a 50 ml medium. Flasks were inoculated with 1 ml cultures pregrown. Then, isolates were incubated at 30°C under agitation (180 rpm) for 10–15 days (depending on the strain). Samples were taken at intervals from shake flasks. The isolated yeast was also grown on Potato Dextrose Agar (PDA) in order to observe larger colonies for better study. Growth was assessed by measuring the optical density (OD) at 600 nm. Three independent experiments were carried out for each yeast strain.

### 2.3. Biomass Growth during the Biodegradation Process

Biomass concentration was estimated from the absorbance of appropriately diluted culture medium at 600 nm (Ultrospec 3300 pro UV/Visible) according to the predetermined correlation between optical density and dry weight of biomass. During the biodegradation assays, the number of viable yeasts was measured by counting of heterotrophic microbes in Petri dishes, expressing the result as colony-forming units (CFU) per ml. With a sterile pipette, 1 ml of sample was taken from the culture and a series of 1 : 10 dilutions made in NaCl solution at 0.9%. Each dilution was analysed in duplicate: 1 ml of sample to be analysed was placed on Petri dishes. Then, 20 ml of previously sterilized culture medium was poured onto the Petri dishes, tempered at 60°C, and gently stirred to complete the homogenization. The mixture was cooled until complete solidification and then incubated at 30°C for 72 h. The total number of microorganisms was determined by multiplying the number of CFU by the corresponding dilution factor. The count was made in an automatic colony counter (Countermat, Flash IUL Instruments). All experiments were performed in triplicate with appropriate controls.

### 2.4. Cell Surface Hydrophobicity Test

Cell surface hydrophobicity was measured by microbial adherence to hexadecane according to the method of Rosenberg et al. [17]. Yeast strains were grown in 50 ml basal medium containing hexadecane (1.5%) or glucose (20 mM) as sole carbon source. Yeast cells were washed twice and resuspended in PUM buffer, pH 7.1 (22.2 g K_2_HPO_4_·3H_2_O, 7.26 g KH_2_PO_4_, 1.8 g urea, 0.2 g MgSO_4_·7H_2_O, and distilled water of 1000 ml), to an initial absorbance at 550 nm of 0.5–0.6. The cell suspension (1.2 ml) with hexadecane (0.2 ml) was vortexed in a test tube vigorously for 2 min and left at room temperature for 1 h. The optical density of the bottom aqueous phase was then measured at 550 nm. Hydrophobicity was expressed as the percentage of adherence to the hydrocarbon which was calculated using the following:

(1) % Hydrophobicity=100×1−OD of the aqueous phaseOD of the initial cell suspension.

Hydrophobicity of the yeasts grown on glucose was the reference of hydrophobicity.

### 2.5. Tests of Hydrocarbon Tolerance and Biodegradation Studies

In order to determine the degradation of* n*-alkanes present in the hydrocarbon-rich wastewater by the selected yeast isolate, the total petroleum hydrocarbon (TPH) was determined by the gravimetric weight loss technique [18]. The analyses of hydrocarbons were carried out after dichloromethane extraction. The aqueous phase sample was removed and put in a sealed flask for subsequent analysis. Then, it was concentrated to approximately 3 ml using a rotary evaporator under reduced pressure in a water bath. Afterwards, it was dissolved in equal volume of dichloromethane and further cleaned through a column filled with florisil (SUPELCLEAN LC-FLORISIL, USA) and then analyzed by gas chromatography, mass spectrometry apparatus. After the evaporation of the solvent, the amount of residual TPH was determined by gravimetric methods after dichloromethane evaporation by simple distillation at 60°C [18]. Hydrocarbon content was determined using an extraction of hydrocarbon according to the standard method for oil gravimetric determination. The degree of biodegradation was calculated as [1 − (*Xo* − *X*1)/*Xo*] 100% [%], where *Xo* is initial amount of hydrocarbon and *X*1 is amount of hydrocarbon after biodegradation. 

The ability of the selected strains to utilize hydrocarbons as the sole carbon source was tested by growing yeasts strains in MM with different hydrocarbons in separate flasks. The biodegradation assay was carried out over 12 days in six 250 ml Erlenmeyer flasks (corresponding to 0, 4, 6, 9, and 12 days of experiment, resp.), with each flask containing 50 ml of the mineral medium and 10% of acclimated inoculum under aseptic conditions. For the induction and hydrocarbon degradation studies using nonproliferating cells, strains HC1 and HC4 were grown in Erlenmeyer flasks containing 50 ml MM with 1% hydrocarbon compounds as the sole carbon and energy source and were incubated in a rotary shaker (180 rpm) at 30°C. The cells were washed twice with sterile 50 mM potassium phosphate buffer, pH 7.6. Growth on individual substrate was removed periodically to assess the concentration of following changes in optical density at 600 nm of washed cells against biotic (without a substrate) and sterile (without bacteria) controls and tested for its bioremediation capacity* in situ* mesocosm to degrade multiple substrates after 12-day incubation period.

### 2.6. Hydrocarbon Analysis

The evaluation of the hydrocarbons biodegradation of the contaminated wastewater effluent by yeast strains was carried out during the process of 12 days using gas chromatography with mass spectrometry GC/MS. After 12 days of incubation, the undegraded alkane hydrocarbon residue was extracted twice with equal volumes of dichloromethane and the aromatic residue with toluene. One microlitre of the alkane fraction (dissolved in dichloromethane) was analyzed by gas chromatography analysis GC/MS (Shimadzu, Model 5975B inert MSD) using a HP-5MS chromatographic column (5% phenyl Methyl Siloxane) of size 30 m × 0.25 mm × 0.25 *µ*m. The temperature was programmed to vary linearly from 70°C to 230°C at the rate of 20°C min^−1^ then from 230°C to 300°C at 40°C min^−1^ and 10 min at 300°C. Samples of 1.0 *µ*l were injected into the GC/MS, operating in the splitless mode with an injector temperature of 250°C, and Helium was the carrier gas.

Similarly, the aromatic fraction was dissolved in toluene and 1 ml was analyzed by GC/MS using a 30-m long HP-5.MS (0.25 mm film thickness) column. During analysis, the injector and detector of GC/MS were maintained at 300°C and the oven temperature was programmed to rise from 150°C to 300°C with an increase of 5°C per minute and then held at 300°C for 5 min.

The highest resolution chromatographic peaks were scanned to find their corresponding mass fragmentation profile. Compounds were characterized based on similarities between their mass spectrum and those presented by Wiley Compounds Library. Control peak-areas were used as a point of reference for the remaining compounds (100%) in the untreated system. Sample peak-areas were reported as a percentage of control peak-areas. Individual compounds present in the alkane and aromatic fractions were determined by matching the retention times with authentic standards (the* n*-alkanes: C8, C10, C11, C12, C13, C14, C17, C18, C19, C20, C21, C22, C23, C24, C25, C28, and C30 and the aromatic hydrocarbon standards fluoranthene, pyrene, phenanthrene, and anthracene were obtained from Sigma-Aldrich, USA).

### 2.7. Identification and Phylogenetic Affiliation of the Yeast Strains

Genomic DNA of the strains HC1 and HC4 was isolated using a protocol described by Masneuf-Pomarède et al. (2007) [19]. The primers ITS1 (5′-TCCGTAGGTGAACCTGCGG-3′) and ITS4 (5′-TCCTCCGCTTATTGATATGC-3′) described by White et al. (1990) [20] were used to amplify the 5.8S ITS region of strain HC4. The D1/D2 domain of 26S rDNA gene of strain HC1 was amplified under the same conditions with primers NL1 (5′-GCATATCAATAAGCGGAGGAAAAG-3′) and NL4 (5′-GGTCCGTGTTTCAAGACGG-3′) [21]. The PCR reaction was performed on a Thermocycler GeneAmp PCR System 9700 (Applied Biosystems) in a 50 *µ*l reaction mixture containing 5x GoTaq reaction buffer (Promega), 0.25 mM each dNTP, 0.2 *µ*M each primer, 50 ng DNA template, and 2.5 U GoTaq DNA polymerase (Promega). The PCR program was as follows: denatured by heating for 2 min at 94°C and subjected to 30 cycles for 30 s at 94°C, 45 s at 58°C, and 1 min 45 s at 72°C, this was followed by a final elongation step for 10 min at 72°C. PCR products were purified with illustra GFX PCR DNA and Gel Band Purification kit (Amersham Biosciences, GE Healthcare) according to the manufacturer's protocol. DNA sequencing of PCR products was performed using a BigDye Terminator v3.1 Cycle Sequencing Kit on the ABI PRISM 3100-Avant Genetic Analyser (Applied Biosystems). Target sequences were analyzed using BLAST online (https://blast.ncbi.nlm.nih.gov/) [22]. The related sequences were collected and aligned using the MUSCLE software [23, 24]. A phylogenetic tree was constructed using the neighbor-joining method in MEGA version 4.1. Yeast species were identified based on the phylogenetic analysis results [25]. The topology of the distance tree was tested by resampling data with 1000 bootstraps to provide confidence estimates. Nucleotide sequences obtained in this work were deposited in the NCBI GenBank data library.

## 3. Results and Discussion

### 3.1. Isolation and Screening of Hydrocarbons-Degrading Microorganisms

**Figure 1:**
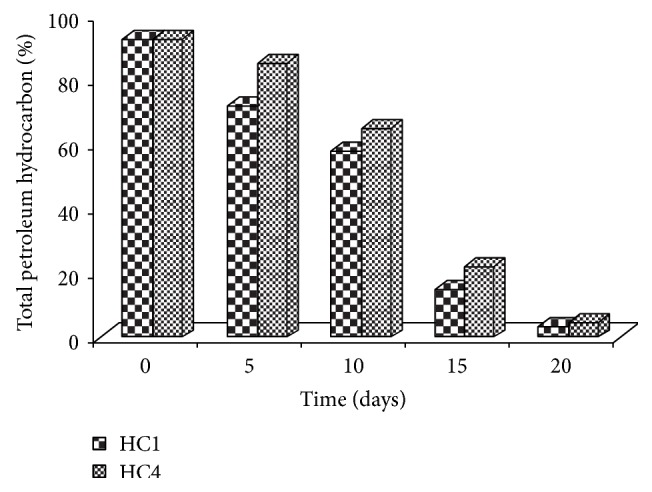
Biodegradation efficiency of yeast strains.

To isolate different hydrocarbon-degrading yeasts, enrichment culture methods were used according to protocol described in Material and Methods. The enrichment procedure for obtaining contaminated wastewater hydrocarbon-degrading microbes was performed in multiple cycles to ensure that the microbes which were obtained at the end of the enrichment cycle were capable of utilizing the petroleum compounds rather than just tolerating it. Five yeast strains were isolated from enrichment cultures that were established at 30°C for 2 weeks. Two of the isolated strains that showed higher growth rate on crude oil were selected from the five isolates for further study. These isolates showed a varying degradation profile for the total petroleum hydrocarbon [TPH] of the petroleum contaminated wastewater. It varied from 29.5% to 95% (Figure 1). In our present work, we report that yeasts* Candida tropicalis* and* Trichosporon asahii* could efficiently degrade total petroleum hydrocarbon removal about 97% and 95%, respectively, over a period of 20 days. According to many authors, bacteria have been described as being more efficient hydrocarbon degraders than yeast or at least that bacteria are more commonly used as a test microorganism [26–28]. Many other investigators have reported the involvement of bacteria and yeast in crude oil biodegradation [12, 29]. On the contrary, there is scanty information that yeasts are better hydrocarbon degraders than bacteria [11, 30]. For that reason, growth ability of two selected strains (HC1 and HC4) was tested in an enrichment liquid medium.

### 3.2. Phylogenetic Analyses of the Yeast-Degrading Hydrocarbon

**Figure 2:**
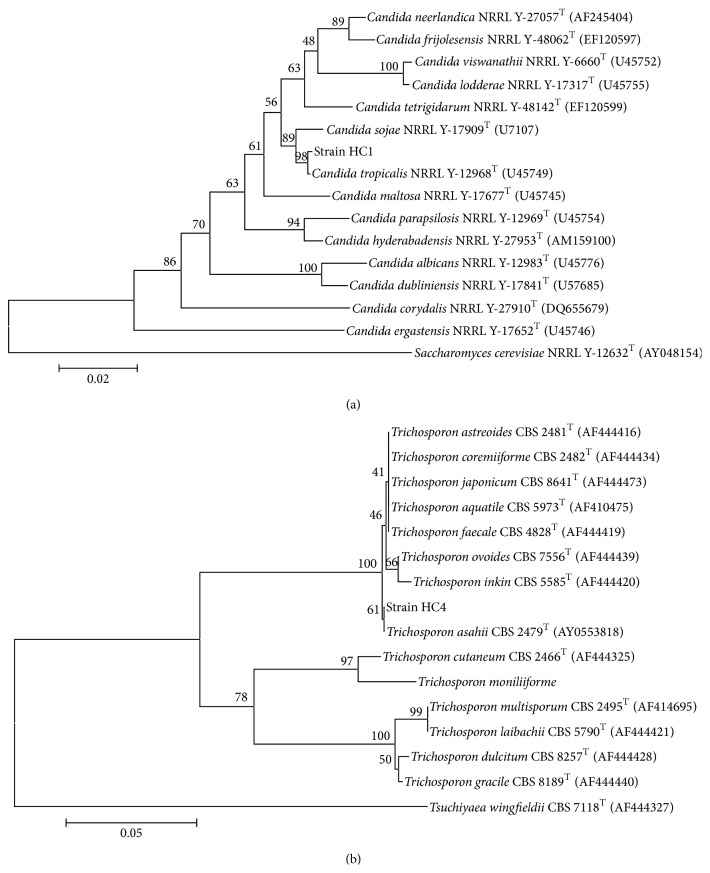
Phylogenetic dendogram data showing the taxonomic location of strains HC1 and HC4. (a) Neighbour-joining phylogenetic dendogram based on sequences of the D1/D2 domain of the 26S rRNA gene of HC1 and related taxa. Bootstrap values are given at the nodes. Scale bar represents the substitution percentage.* Saccharomyces cerevisiae* NRRL Y-12632^T^ was used as outgroup. GenBank accession numbers follow species name in parenthesis. (b) Phylogenetic dendogram obtained by neighbour-joining analysis of the 5.8S-ITS sequence of HC4 and related taxa. Bootstrap values, determined from 100 replicates, are shown at branch nodes. Scale bar represents the substitution percentage. The outgroup we used was* Tsuchiyaea wingfieldii* CBS 7118^T^. GenBank accession numbers follow species name in parenthesis.

To analyse the phylogenetic position of the yeast isolates, the 26S rRNA gene of isolate HC1 and the 5.8S-ITS noncoding regions of the rDNA of the isolate HC4 were amplified, sequenced, and compared with those available in the GenBank nucleotides sequences database. Percentage similarity values were obtained after pairwise alignment of the sequences of the 16S rDNA of the yeast strains and EMBL database sequences. The sequences giving the highest scores were retried to construct the phylogenetics dendograms. Sequence comparison demonstrated the affiliation of the strain HC1 to the genus* Candida*. The phylogenetic analysis based on the D1/D2 sequences showed that strain HC1 belongs to ascomycetous yeasts and indicated that strain HC1 is closely associated with the speciesof* Candida tropicalis* in Figure 2(a). The determined sequences displayed similarities of 99.4%, 99%, 98.8%, and 99% to* Candida sojae*,* Candida tetrigidarum*,* Candida maltosa,* and* Candida neerlandica*, respectively [31, 32].

Identification of the yeast strain HC4 was performed by amplifying the 5.8-ITS rRNA. Compared to the 5.8-ITS rDNA sequence analysis, it was observed that the strain HC4 has identical sequence to* Trichosporon asahii* CBS 2479^T^. Basidiomycetous yeast HC4 strain was clustered closely with* Trichosporon asahii* and showed also more than 99% similarity between the 16s rRNA gene sequences with the* Trichosporon astreoides *and* Trichosporon coremiiforme. Basidiomycetous* yeast HC4 also shared similarity of about 99.7%, 99%, and 99.8% identities with* Trichosporon faecale, Trichosporon japonicum,* and* Trichosporon aquatile, *respectively [33]. Phylogenetic relationships of the sequence of 5.8S ITS region of the yeast strain HC4 are shown in Figure 2(b) and placed this isolate in the Ovoides clade of the genus* Trichosporon*. The nucleotide sequences for the two yeast strains (HC1 and HC4) determined in this study have been deposited in the GenBank under the accession numbers JN088216 and JN088217, respectively.* Candida tropicalis* and* Trichosporon asahii* are a well-known species of microorganisms to degrade hydrocarbons and have been used widely for petroleum remediation [34, 35].

### 3.3. Cell Surface Hydrophobicity

**Figure 3:**
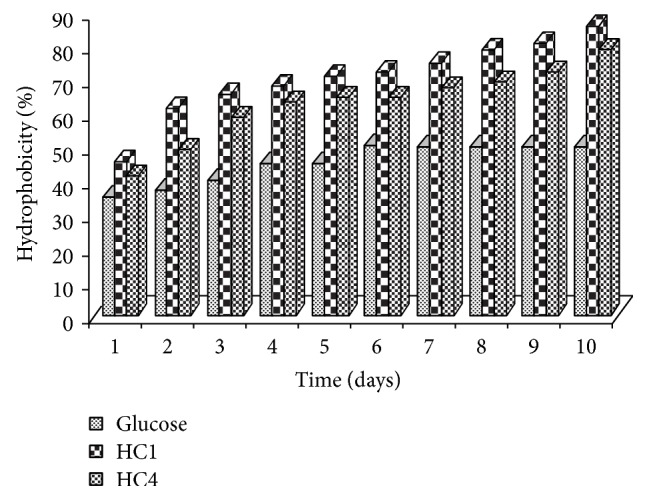
Hydrophobicity of yeast strains isolates during growth in mineral medium with 1.5% hexadecane or glucose as a sole carbon source. □ after growth on glucose; ■, after growth on hexadecane. Values are the mean of three separate experiments ± s.d.

Results for cell surface hydrophobicity when the strains were grown on hexadecane and glucose are shown in Figure 3. As it can be seen, cell hydrophobicity of tested strains remained unchanged during growth on glucose. Cell hydrophobicity reached approximately 78 and 85.4% with* Trichosporon *and* Candida* strains, respectively. During our studies, and for both yeast strains, we noticed that the maximal increase in hydrophobicity is correlated with the maximum biodegradation of hexadecane (or TPH). A direct relationship was found between both cell hydrophobicity of yeast strains and petroleum hydrocarbon biodegradation (Figures 1–3). Based on data obtained from growth and cell hydrophobicity, cell surface hydrophobicity could be proposed as the most probable mechanism for hexadecane uptake by the tested yeast strains. Increase in cell hydrophobicity by both yeast strains during growth on hexadecane indicated that the strains could employ biosurfactant-mediated alkane uptake. In fact, Prabhu and Phale suggest that a biosurfactant production and a cell surface hydrophobicity play an important role in hydrocarbon uptake [36]. In this case, the cell contact with hydrophobic compounds is a requirement because the first step in aromatic or aliphatic hydrocarbon degradation is the introduction of molecular oxygen into the molecules by cell-associated oxygenases [37]. Cell hydrophobicity can be considered one of the important factors controlling hydrocarbon assimilation. The yeast strain* Trichosporon asahii* isolated from petroleum contaminated soil in India by Chandran and Das produced biosurfactant in mineral salt media containing diesel oil as the carbon source and degraded diesel oil (95%) over a period of 10 days [34]. An extracellular bioemulsifier was isolated from* Candida tropicalis* and this composed stable hexadecane-in-water emulsions [38].

### 3.4. Microbial Growth on Hydrocarbon Compounds

**Figure 4:**
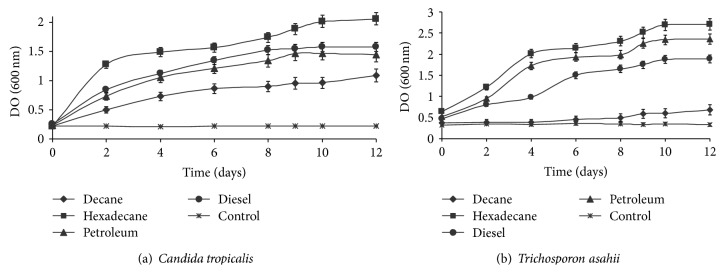
Growth of selected yeasts on mineral medium with some alkanes (1% w/v) as the sole carbon source with respect to uninoculated controls over 12 days of degradation. (a)* Candida tropicalis,* (b)* Trichosporon asahii.*

The extent of oil-related compounds degradation and the time for complete hydrocarbon compound degradation varied as a function of yeast strains. In addition to their capacity to grow on hexadecane, strains can also grow on some refinery subproduct such as crude oil, diesel fuel, and lubricating oil (Figure 4). Hexadecane was the best substrate to support yeast strains growth. However, the growth of strain belonging to the genus* Trichosporon* in the presence of diesel fuel showed extended long degradation time (Figure 4(b)). Diesel oil is a medium distillate of petroleum containing* n*-alkanes, branched alkanes, and small concentrations of aromatic polycyclic compounds. Yeast strains were able of utilizing a wide range of hydrocarbons, with a preference for alkanes with intermediate carbon chain lengths. Measurement of growth attributes of the isolates using* n*-hexadecane, diesel oil, and crude oil as substrates showed that the yeast strains were better while using of hydrocarbon substrates. Thus, the biodegradation of mixtures of substrates is an important way in biological treatment of petroleum contaminated wastewater. In fact, petroleum is found typically as complex mixtures in the environment, containing distinct petroleum constituents, such as alkanes (linear, branched, and cyclic), PAHs, heterocyclic aromatic compounds, and asphaltenes. Yeasts are relatively rarely isolated from crude oil or hydrocarbon sources. Moreover, yeasts are capable of using a wide range of different carbon sources [29], though only few studies have been carried out on the potential use of yeast on oil-contaminated sites [39].

The decreased growth pattern in short chain could be attributed to the toxicity of these alkanes to cell membranes [40]. On the other hand, while growth on long chain alkanes could be limited by decreased bioavailability of the substrate, the ability of the yeast isolates to use long chain *n*-alkanes in preference to shorter-chain, ones below C8–C10, could be attributed to reduced toxicity and higher growth yields obtainable from those substrates. Substrates growth could either be due to the constitutive nature of hydrocarbon assimilating capabilities in the isolates or reflect the adaptation of the yeast strains due to previous exposure to exogenous hydrocarbons. It may also indicate the ability of the yeast strains to emulsify hydrocarbons, which is a major factor for hydrocarbon uptake and assimilation.

**Figure 5:**
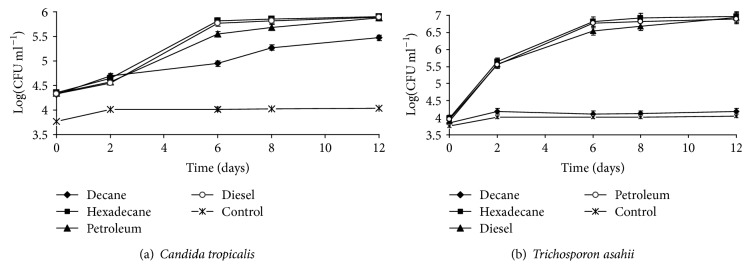
Yeast cell count during 12 days. (a)* Candida tropicalis*, (b)* Trichosporon asahii.* Each point represents mean ± S.E of triple assays.

These results were confirmed also by viable cells enumeration, indicating positive growth with the three substrates shown in Figure 5. These findings suggest that the capacity to degrade crude oil by yeast strains is higher than that by diesel fuel. However, the major compound of diesel fuel is aromatic hydrocarbons. These compounds are more recalcitrant than aliphatic hydrocarbon present in crude oil and lubricating oil [40].

### 3.5. Degradation of the Alkane and Aromatic Fractions by Yeast Strains

**Figure 6:**
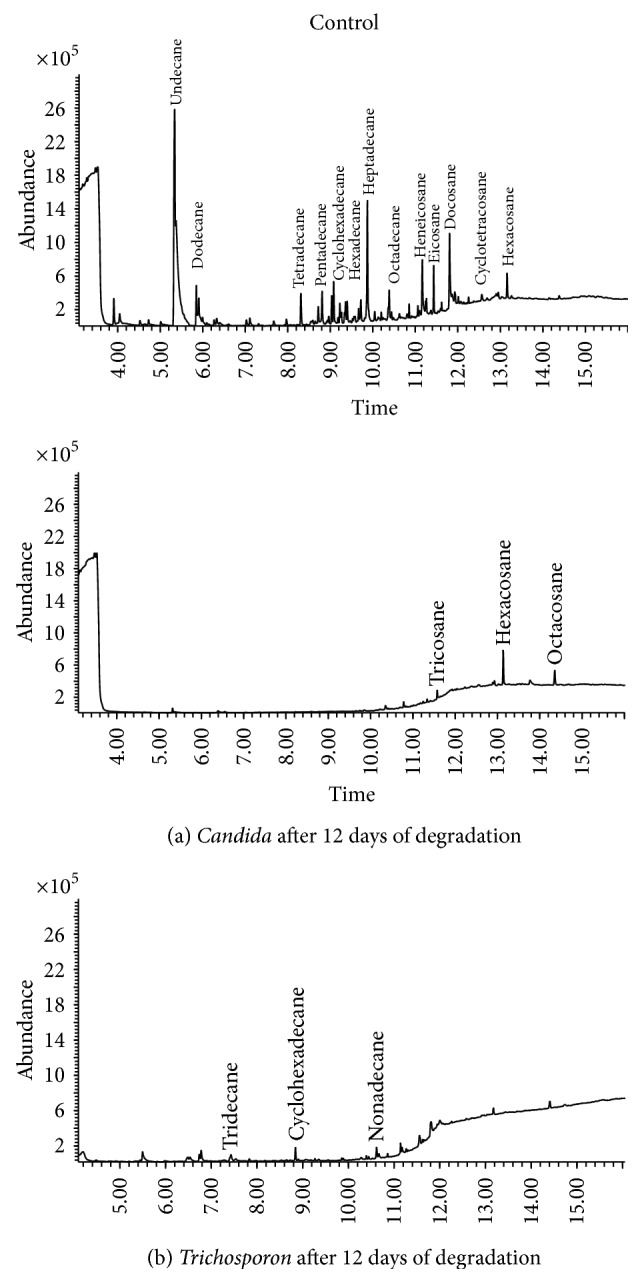
Gas chromatogram showing the biodegradation of alkane fraction of rich-hydrocarbon wastewater from Tunisian refinery by* Candida tropicalis *(a) and* Trichosporon asahii *(b) compared to the control.

**Figure 7:**
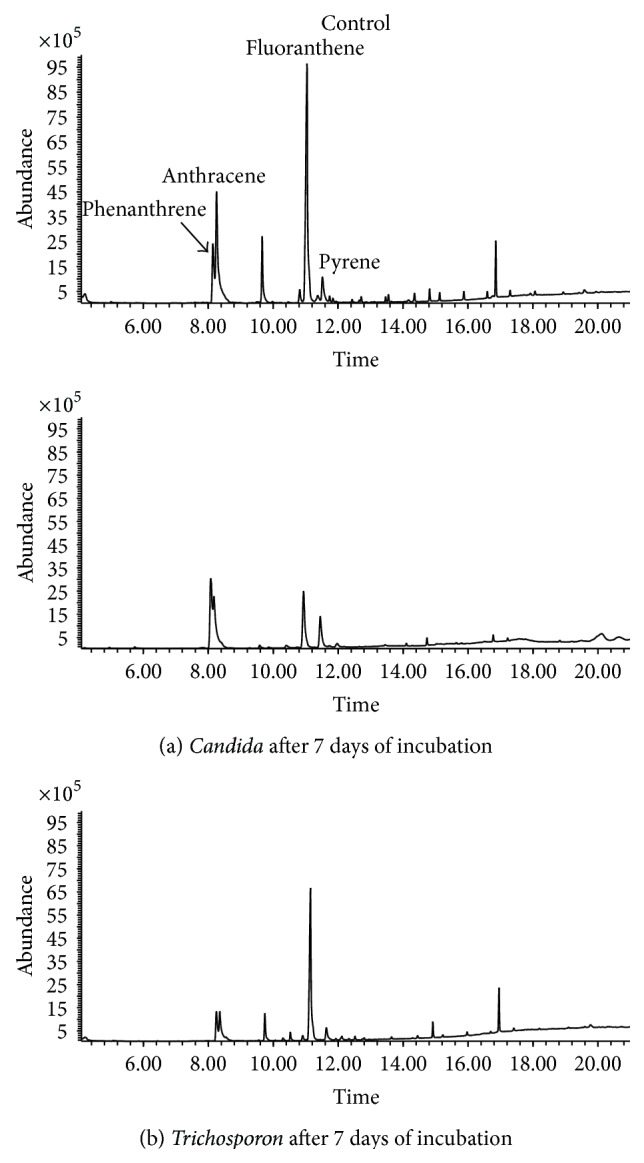
Gas chromatogram showing the biotransformation of the aromatic fraction of rich-hydrocarbon wastewater from Tunisian refinery by* Candida tropicalis* (a) and* Trichosporon asahii* (b) compared to the control.

The degradation of the alkane and aromatic hydrocarbons by* Candida* and* Trichosporon* species was analyzed by gas chromatography coupled to mass spectrometry (GC/MS). From Figure 6, the strains depicted efficient utilization of the alkane fraction compared to the untreated control. Analysis by GC/MS revealed that both isolates were capable of degrading the aliphatic fractions, which showed that all the detectable hydrocarbons peaks were completely utilized within 12 days by the two yeast isolates. The isolate yeast strains were capable of degrading the entire range of* n*-alkanes on the medium containing petroleum hydrocarbons, from undecane (C11) to hexacosane (C26), as the sole carbon source and energy. The hydrocarbon utilization pattern of yeast strains indicated that the growth was less on short chain alkanes like octane, decane, and undecane. However, it started increasing from dodecane with a maximum growth being observed on heptadecane, octadecane, and nonadecane after which the growth was again comparatively reduced with eicosane, tricosane, tetracosane, and hexacosane (Figure 6). Both short and long chain hydrocarbons of the industrial wastewater refinery were highly susceptible to be attacked by the yeast strains, probably due to the fact that they have a very efficient degradative enzyme system. The strains also showed considerable utilization of components from aromatic fraction as depicted in Figure 7. While* Candida* and* Trichosporon* species were capable of efficient degradation of alkanes as the sole carbon source, they were unable to completely degrade aromatic hydrocarbons as the sole carbon source. Aromatic compounds proved to be resistant to biodegradation and we observed that* Trichosporon* can assimilate phenanthrene, anthracene, and fluoranthene by 47%, 73%, and 31.4%, respectively, within 7 days.

The yeast strain* Candida* species HC1 was capable of degrading a mixture of low and high molecular weight of aromatic hydrocarbons and a degradation efficiency was observed with anthracene (51%) and fluoranthene (74%) at the end of 7 days.

Aromatic hydrocarbons are known to be more resistant to biodegradation than aliphatic compounds and are often a serious problem during bioremediation processes [18, 41]. However, the* n*-alkanes are the most biodegradable petroleum hydrocarbons and are attacked by more microbial species than aromatic or naphthenic compounds. The oxidation of alkanes was considerably more profound than that of aromatic hydrocarbons. This finding is in agreement with other reports where hydrocarbon-degrading microbes have shown preferential degradation of alkanes to aromatics and NSO-asphaltene fractions [18, 42]. However, as expected, the results obtained in the laboratory may not scale up to the natural environment thus we must analyze the behavior of these strains in mesocosm experiments to determine how they may act in the natural environment for the bioremediation of hydrocarbon-polluted wastewater.

## 4. Conclusion

Our study focused on the yeast strains isolated from enrichment culture of the contaminated industrial effluent from Tunisian petroleum refinery wastewater, identified as* Candida tropicalis *and* Trichosporon asahii* which developed adaptation mechanisms to survive in the harsh environments of that refinery. A direct relationship was found between both cell hydrophobicity of the yeast strains and crude oil biodegradation. These strains have high levels of crude oil degradation and sufficient growth on some aliphatic hydrocarbons. In this study, the isolated yeasts have been shown to degrade a wide range of hydrocarbons and completely metabolize* n*-alkanes. On the other hand, they were unable to completely degrade aromatic hydrocarbons (phenanthrene, anthracene, fluoranthene, and pyrene) as the sole carbon source. From the data presented in this study, it can be concluded that the investigated strains* Candida* and* Trichosporon* could be considered as good prospects for their application in bioremediation of hydrocarbon contaminated environment and improvement of hydrocarbon removing treatment of industrial wastewater.
